# In Vitro Investigation of the Cytotoxic and Antioxidant Activities of *Ardisia polycephala, Iresine herbstii,* and *Oenanthe javanica* Extracts With Potential Applications in Breast Cancer Therapy

**DOI:** 10.1155/adpp/1234439

**Published:** 2025-08-08

**Authors:** Nuntika Prommee, Ubonwan Saesiw, Thana Juckmeta, Pakakrong Thongdeeying, Kitiya Yangthaworn, Bhanuz Dechayont, Jitpisute Chunthorng-Orn, Pathompong Phuaklee, Tassanee Ongtanasup, Onmanee Prajuabjinda

**Affiliations:** ^1^Division of Applied Thai Traditional Medicine, Faculty of Public Health, Naresuan University, Phitsanulok 65000, Thailand; ^2^Department of Applied Thai Traditional Medicine, Faculty of Medicine, Thammasat University, Pathum Thani 12120, Thailand; ^3^Division of Integrative Medicine, Chulabhorn International College of Medicine, Thammasat University (Rangsit Campus), Pathum Thani 12120, Thailand; ^4^Faculty of Allied Health Sciences, Burapha University, Chonburi 20131, Thailand; ^5^Department of Applied Thai Traditional Medicine, School of Medicine, Walailak University, Nakhon Si Thammarat 80160, Thailand

**Keywords:** antiestrogen, *Ardisia polycephala*, breast cancer, *Iresine herbstii*, *Oenanthe javanica*

## Abstract

*Ardisia polycephala, Iresine herbstii,* and *Oenanthe javanica* are commonly used to nourish women's blood and treat reproductive system diseases, according to Thai Traditional Medicine (TTM) scriptures. This study explores the biological activities of these herbs as documented in TTMs. Both water and ethanol extracts of the three herbs were examined for their antioxidant activities using DPPH, FRAP, and NO assays. Additionally, their antiestrogen and cytotoxic effects were investigated, focusing on breast cancer cell lines (T47D and MCF-7). Chemical analysis was conducted using the GC–MS technique, with reference data obtained from the National Institute of Standards and Technology (NIST) library. All herbs demonstrated good antioxidant activity. The water extract of *Ardisia polycephala* (AP^∗∗^) exhibited the highest activity in the DPPH assay (EC_50_ = 7.09 ± 1.47 μg/mL), which correlated with the FRAP results (358.52 ± 1.07 mg Trolox equivalent per gram). The ethanol extract of *Oenanthe javanica* (OJ^∗^) specifically showed cytotoxicity against both T47D and MCF-7 cell lines (IC_50_ = 38.06 ± 1.52 and 39.93 ± 0.36 μg/mL, respectively) and demonstrated antiestrogen activity by inhibiting the growth of T47D cells by 99% at 1.3 μg/mL when stimulated with 100 pM estradiol. GC–MS analysis identified numerous compounds that support the observed biological activities, including antioxidant and cytotoxic effects. In summary, the three herbs demonstrated antioxidant, cytotoxic, and antiestrogen activities consistent with the chemical compounds identified in the GC–MS analysis. These findings suggest the potential use of these herbs in future breast cancer treatments.

## 1. Introduction

*Ardisia polycephala* (AP) Wall. is a shrub or tree that grows primarily in subtropical regions, including Assam, South-Central China, Laos, Myanmar, and Thailand [1]. This Thai medicinal plant is traditionally used to reduce fever and to treat diarrhea, urticaria, and skin diseases [2]. The ethanol extract of AP has been studied for its antiproliferative activity against the SKBR3 human breast adenocarcinoma cell line using an MTT assay [3]. Previous studies have reported the 2,2-diphenyl-1-picrylhydrazyl (DPPH) and FRAP values of the methanol extract of the fruit [4]. Chemical analysis of AP has identified amyrin and rapanone, both of which exhibit antibacterial properties. Additionally, the ripe fruits, which are dark purple to nearly black, contain anthocyanin, a compound with antioxidant effects [5]. The fruit's growth stage affects the total phenolic content, anthocyanin levels, and antioxidant capacity. Studies indicate that as ripening progresses, phenolic content and antioxidant activity decline, while anthocyanin content increases [6].

*Iresine herbstii* (IH) Hook., commonly known as bloodleaf, is native to regions including New Zealand, Australia, and South America. It belongs to the family Amaranthaceae and is known for its small, vividly colored leaves. In addition to its ornamental value, IH is widely used in traditional medicine. In Brazil, its leaves are used for wound healing and exhibit anticancer and antipyretic properties [7]. In northern Peru, the plant is applied externally to treat skin conditions such as eczema, sores, and pimples [8]. In India, it is used as an astringent, diuretic, and spasmolytic, and in the treatment of conditions such as whooping cough and hemicranias, due to its antimicrobial properties [9]. In Ecuador, decoctions of the leaves and flowers are used for fever relief, relaxation, and kidney disorders [10]. Research indicates that IH possesses anti-inflammatory, cytotoxic, and apoptotic properties. However, the betacyanin compounds found in the Amaranthaceae family have demonstrated only low antioxidant activity [11, 12].

*Oenanthe javanica* (OJ) (Blume) DC. (Apiaceae) is a small perennial herb that has been cultivated in tropical and temperate regions of Asia for millennia. Traditionally, it has been used as a folk remedy for a wide range of diseases. In China, OJ has been noted for its hepatoprotective [13], anti-inflammatory [14, 15], immune-enhancing [16], and antiviral properties [17]. Phytochemical analyses have identified several bioactive compounds in OJ, including coumarins, flavonoids, flavonoid glycosides, and polyphenols [18]. Toxicological studies have shown that OJ does not exhibit acute or genetic toxicity [19, 20].

According to Thai Traditional Medicine (TTM) scriptures, these three herbs—characterized by their hot, spicy taste—are used to treat female reproductive system disorders. Based on the Tri-Dhatu theory of elemental balance, these herbs influence the Vata (Wind), Pitta (Fire), and Semha (Water) elements [21]. The Vata element, composed of air and space, distributes heat and cold throughout the body and maintains physiological balance [22]. The hot, spicy taste is believed to restore Vata balance, which is associated with the circulatory system. Some scientific reports have found IH to be a postlabor tonic [23], and OJ has demonstrated antioxidant activity [24]. However, scientific evidence supporting the biological efficacy of these herbs in breast cancer treatment remains limited.

Therefore, the objective of this study was to investigate the biological activities of these selected medicinal plants. Both aqueous and ethanolic extracts of AP, IH, and OJ were evaluated for their antioxidant properties using DPPH, FRAP, and nitric oxide (NO) scavenging assays. Additionally, their antiestrogenic and cytotoxic effects were assessed against breast cancer cell lines T47D and MCF-7. Chemical profiling was conducted using gas chromatography–mass spectrometry (GC–MS) analysis.

## 2. Materials and Methods

### 2.1. Chemicals, Reagents, and Instruments

All chemicals and reagents used in this study were of analytical grade. DPPH and butylated hydroxytoluene (BHT) were purchased from Fluka (Germany). Sulforhodamine B sodium salt (SRB) [C_27_H_29_N_2_NaO_7_S_2_], tris (hydroxymethyl)aminomethane [NH_2_C(CH_2_OH)_3_], insulin solution from bovine pancreas, estradiol, genistein, tamoxifen, and Trolox were obtained from Sigma-Aldrich (USA). Trichloroacetic acid (TCA) [Cl_3_CCOOH], absolute ethanol, and acetic acid were purchased from Merck (Germany). Minimum essential medium (MEM), Roswell Park Memorial Institute (RPMI) medium, fetal bovine serum (FBS), trypan blue stain (0.4%), kanamycin (1%), insulin solution (0.1%), sodium pyruvate (1 mM), and trypsin-EDTA were purchased from Gibco (USA). Hydrochloric acid and isopropanol were purchased from RCI Labscan (Thailand). Phosphate-buffered saline (PBS) was obtained from Biochrom (Germany), and Alamar blue was purchased from Bio-Rad (UK). Kanamycin sulfate (100X) was obtained from Gibco (China). Milli-Q water was prepared using the PURELAB Classic-US system (ELGA, UK). A Multiskan GO microplate reader was used for absorbance measurements (Thermo Scientific, Finland), and a Varioskan LUX multimode microplate reader was used for fluorescence measurements (Thermo Fisher Scientific Inc., USA).

### 2.2. Plant Materials and Preparations

AP, IH, and OJ were purchased from Charoensuk Osod, a Thai herbal medicine store in Nakorn Pathom Province, Thailand. Botanical authentication was performed, and the voucher specimens were deposited at the Thai Traditional Medicine Herbarium, under the Thai Traditional Medicine Research Institute, Ministry of Public Health, Thailand.

The plant materials were washed, dried, and ground into small pieces for extraction. Water and ethanol extracts were prepared according to a previously described method [25]. Crude extracts were stored at −20°C until use.

For in vitro experiments, ethanolic extracts were dissolved in sterile DMSO at a concentration of 10 mg/mL. Aqueous extracts were dissolved in sterile water at the same concentration and filtered through a 0.22-μm sterile syringe filter. All samples were further diluted to appropriate concentrations using cell culture media.

### 2.3. Antioxidant Assays (DPPH, FRAP, and NO)

The DPPH radical scavenging assay was conducted following a previously published method [21], while the FRAP assay followed the protocol outlined in [26]. Ethanolic extract stock solutions (1 mg/mL) were prepared in absolute ethanol; aqueous extracts were prepared in distilled water. All samples were serially diluted into four concentrations for the DPPH assay. BHT was used as the positive control. Absorbance was measured at 520 nm using a microplate reader. Radical scavenging activity was calculated using the following formula:

(1) $$\begin{array}{c} t\text{he percentage of inhibition}=\frac{\text{Abs}.\text{Control}-\text{Abs}.\text{Sample}}{\text{Abs}.\text{Control}}\times 100. \end{array}$$

A dose–response curve was generated, and EC_50_ values were calculated using GraphPad software.

For the FRAP assay, a working solution was prepared at a 10:1:1 ratio of acetate buffer, TPTZ solution, and FeCl_3_·6H_2_O, respectively. Trolox, dissolved in absolute ethanol, was used as a standard to generate a calibration curve. After 8 min of incubation, absorbance was measured at 593 nm. Results are expressed in mg Trolox equivalents per gram of extract (mg TE/g extract).

The NO scavenging assay was adapted from a previously published method [27]. Samples were serially diluted into at least 4 concentrations. After reaction with Griess reagent (1% sulphanilamide and 0.1% N-naphthyl ethylenediamine dihydrochloride in 2% H_3_PO_4_), absorbance was measured at 550 nm.

### 2.4. In Vitro Cytotoxic Activity

Cytotoxicity was evaluated using the SRB assay, modified from previously reported methods [28, 29]. The breast cancer cell lines MCF-7 (ATCC# HTB-22) and T47D (ATCC# HTB-133) were cultured in MEM and RPMI media, respectively. MCF-7 cells were maintained in MEM supplemented with 10% FBS, 1% of 1 mM NEAA, 1% of 1 mM sodium pyruvate, and 1% of P/S. T47D cells were maintained in RPMI medium supplemented with 10% of FBS, 1% of P/S, 1% of kanamycin, and 0.1% of insulin solution. All cells were incubated at 37°C in 5% CO_2_ with 95% humidity.

The optimal plating densities of each cell line were calculated based on cell growth profiles. MCF-7 and T47D cells were seeded at 3 × 10^4^ cells/well and 7 × 10^3^ cells/well, respectively. After 24 h, the cells were treated with different concentrations of crude extracts. DMSO was used as a solvent. The plates were incubated for 72 h. Then, the supernatant was removed, and the cells were washed with sterile PBS. Media (200 μL/well) was added and incubated for 72 h.

Cells were fixed by 100 μL/well of ice-cold 40% TCA and incubated at 4°C for 1 h. After that, plates were washed five times with tap water to drain off the nonviable cells and then air-dried. Cells were stained with 0.4% SRB solution in 1% acetic acid (50 μL/well) for 30 min and then washed four times with 1% acetic acid. The protein-bound dye was dissolved using 10 mM Tris base (100 μL/well), and the plates were shaken gently for about 20 min. Optical density (OD) was measured at 492 nm. Cytotoxicity was expressed as a percentage of inhibition using the formula:

(2) $$\begin{array}{c} t\text{he percentage of inhibition}=\frac{\text{OD Control}-\text{OD Sample}}{\text{OD Control}}\times 100. \end{array}$$

The IC_50_ values were calculated using the Prism program. According to National Cancer Institute guidelines, plant extracts with IC_50_ values less than 20 μg/mL are considered active.

### 2.5. In Vitro Antiestrogenic Effect

#### 2.5.1. Cell Proliferation Assay

The cell proliferation assay for estrogenic activity was performed as described in a previous report [30]. Fluorescence was measured at 590 nm with excitation at 550 nm using a Varioskan LUX multimode microplate reader to assess cell concentrations.

#### 2.5.2. Antiestrogenic Activity

Antiestrogenic activity was assessed following the procedure described in a previous report [31]. MCF-7 and T47D cells were seeded at 1.0 × 10^4^ cells/well in 96-well plates containing 90 μL of RPMI phenol red-free medium supplemented with 5% DCC-treated FBS. After 3-h incubation, 5 μL of each test extract at five different concentrations (0.01–100 μg/mL) and 5 μL of 20 nM estradiol (E2) were added per well to a final volume of 100 μL. The plates were incubated at 37°C for 96 h. Tamoxifen (0.00001 to 1 μg/mL) was used as a positive control. Alamar blue was used to evaluate the cell concentrations, and then, it was incubated for 3 h. Fluorescence was measured at 590 nm with excitation at 550 nm.

IEqE values (iEqE50, iEqE10, and iEqE1) were calculated, representing the concentrations needed to suppress the E2 effect to levels equivalent to 50, 10, and 1 pM, respectively.

### 2.6. GC–MS Analysis

The chemical composition of ethanolic extracts of AP^∗^, IH^∗^, and OJ^∗^ was analyzed using a Scion 436-GC system coupled with a single quadruple mass spectrophotometer and equipped with a CP-8410 autosampler and a SCION-5MS fused silica capillary column. Helium was used as the carrier gas at a flow rate of 1 mL/min. Each sample (10 μL) was injected into the capillary column and run for 60 min. The initial temperature was set to 80°C, increased to 250°C at a rate of 5°C/min until 38 min, and then raised to 280°C at 20°C/min. Component identification and %peak areas were determined by comparing spectra with the National Institute of Standards and Technology (NIST) library.

### 2.7. Statistical Analysis

All experiments were performed in triplicate, and data are expressed as mean ± standard error of the mean (SEM). Statistical analysis was conducted using one-way ANOVA followed by Dunnett's multiple comparison test in SPSS software.

## 3. Results and Discussion

Thai traditional doctors analyze the causes of diseases by assessing the balance of three elements, known as Tri-Thart. These elements are Vata (Wind), Pitta (Fire), and Semha (Water) (Endo and Nakamura, 1995). Various herbal medicines are used to restore the balance of these elements [32]. AP, IH, and OJ are herbal medicines traditionally used to treat imbalances in the fire element, which are associated with conditions such as amenorrhea. These herbs have a spicy taste that, according to Thai traditional beliefs, helps restore the fire element.

Traditionally, Thai doctors ground these herbs into a powder, mixed them with hot water, and administered them orally [33]. In some methods, whiskey was used as a solvent. Modern Thai traditional medicine increasingly employs scientific methods to investigate the efficacy of herbal formulations. In this study, we mimicked traditional preparation methods by performing water and ethanolic extractions. Water extracts from all three herbs yielded higher percentages (ranging from 2.6% to 24.5%) than ethanol extracts. AP had the highest yield, while IH had the lowest yield in both types of extractions.

Oxidative stress is closely associated with imbalances in Vata, Pitta, and Semha. Vata, composed of air and space, governs movement and communication within the body. Pitta, embodying fire and water, regulates metabolism and transformation processes. Semha, often linked to Kapha, represents earth and water, contributing to structure and stability. Imbalances in these doshas can lead to oxidative stress, resulting in cellular damage and contributing to various health issues. Thus, maintaining balance among the doshas is essential for maintaining overall health and preventing oxidative damage [34, 35].

In the DPPH assay, a lower EC_50_ value indicates higher antioxidant activity [36]. The fruit extracts of AP^∗∗^ and AP^∗^ demonstrated stronger radical scavenging activity compared to the positive control, BHT, with EC_50_ values of 7.09 μg/mL and 10.95 μg/mL, respectively. This is consistent with previous reports that found methanol extracts of AP fruits are rich in phenolics and exhibit high antioxidant activity [4]. Conversely, the leaf extracts of IHE and IHW exhibited EC_50_ values ranging from 20.43 μg/mL to 22.61 μg/mL. The water extract of OJ fruits had moderate activity (EC_50_ = 34.14 μg/mL), while the ethanol extract showed no activity (> 100 μg/mL).

Results from the FRAP assay were consistent with the DPPH assay. AP^∗∗^ had the highest FRAP value at 358.52 ± 1.07 mg Trolox equivalent per gram, while OJ^∗^ showed the lowest at 17.72 ± 0.85 mg/g. In the NO radical scavenging assay, all extracts showed significant activity compared to the standard drug, acetaminophen (Table 1).

**Figure 1:**
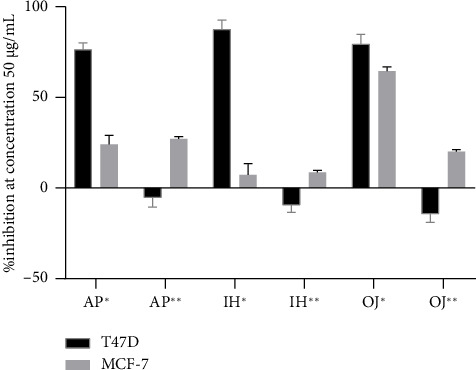
% Inhibition of the extracts from AP, IH, and OJ at concentration 50 μg/mL, mean ± S.D. (*n* = 3) on cytotoxic activity. ^∗^Extract for ethanol maceration; ^∗∗^extract for water decoction.

**Figure 2:**
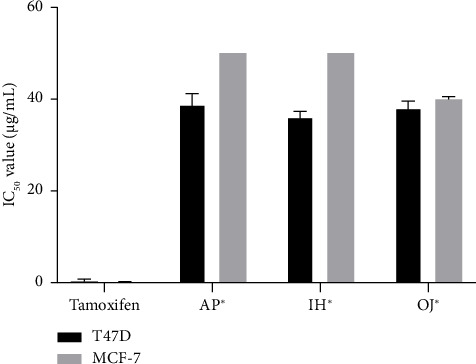
Cytotoxicity of APE, IHE, and OJE against T47D and MCF-7 cell lines; each bar represents a mean of IC_50_ values ± SD (μg/mL) (*n* = 3). ^∗^Extract for ethanol maceration; ^∗∗^extract for water decoction.

Six extracts (both water and ethanol) at 50 μg/mL were screened for in vitro cytotoxicity. As shown in Figure 1, only the ethanol extracts, specifically AP^∗^, IH^∗^, and OJ^∗^, demonstrated significant cytotoxic activity against breast cancer cell lines, with more than 50% inhibition.  Figure 2 presents IC_50_ values for these extracts against the T47D cell line, ranging from 36.10 μg/mL to 38.85 μg/mL. Notably, OJ^∗^ exhibited cytotoxicity against both T47D and MCF-7 cells, with IC_50_ values of 38.06 μg/mL and 39.93 μg/mL, respectively. Although AP has previously demonstrated activity against the HCT116 human colon cancer cell line [37], this is the first report focusing on its effect on breast cancer cell lines.

Antiestrogenic activity against MCF-7 and T47D cell proliferation was investigated for all extracts of AP, IH, and OJ. Estradiol (E2) 100 pM was initially used to enhance cell proliferation, and each extract was tested at 0.01, 0.1, 1, 10, and 100 μg/mL. Tamoxifen, the positive control, suppressed E2-enhanced cell proliferation almost completely (iEqE1) for both cells at concentrations below 1 μg/mL (Table 2). AP^∗^, IH^∗∗^, OJ^∗^, and OJ^∗∗^ inhibited E2-induced proliferation in MCF-7 cells by 50% at concentrations below 2 μg/mL. AP^∗^ and OJ^∗^ showed potent inhibition in both MCF-7 and T47D cells, with iEqE10 values of 0.01 and 3.1 μg/mL for AP^∗^, and 0.12 and 2.47 μg/mL for OJ^∗^, respectively. Furthermore, both AP^∗^ and OJ^∗^ suppressed E2-enhanced proliferation by 99% at iEqE1 concentrations below 4 μg/mL. These results indicate antiestrogenic activity comparable to the positive control, tamoxifen, which was effective below 0.1 μg/mL in both cell lines.

β-Sitosterol, a steroid compound found in AP, IH, and OJ, has been studied for its effects on estrogen-dependent breast cancer cell lines MCF-7 and T47D. It exhibited estrogenic activity in T47D cells but not in MCF-7 cells, and it did not induce chloramphenicol acetyltransferase (CAT) protein expression in HeLa cells cotransfected with mouse estrogen receptor (ER) and an estrogen-responsive CAT reporter gene. These findings suggested that β-sitosterol is not inherently estrogenic but requires metabolic conversion into an active hormonal form to exert such effects [38, 39].

Another study reported that the dietary phytosterol β-sitosterol and the antiestrogen drug tamoxifen affect cell proliferation and ceramide (CER) metabolism in MCF-7 (ER-positive) and MDA-MB-231 (ER-negative). Treatment with β-sitosterol at 16 μM suppressed the growth of both cell lines, whereas tamoxifen at 1 μM inhibited only the MCF-7 cells. Notably, combined treatment of β-sitosterol and tamoxifen resulted in a more pronounced inhibition of cell growth in both cell types, with the most significant effect observed in MDA-MB-231 cells. This synergy is attributed to different mechanisms of CER modulation by each compound, supporting their use as combination therapy for ER-negative breast cancer [40].

An in silico study evaluating pharmacokinetics, molecular docking, and dynamics simulations suggested β-sitosterol is a promising inhibitor of human 17β-hydroxysteroid dehydrogenase type-1 (HSD17B1) [41].

In the GC–MS chromatogram of the AP^∗^ extract, nine peaks were observed, corresponding to compounds such as 2-ethyl-4-hydroxy-5-methyl-3(2H)-furanone, 3-phenylpropionic acid, and β-sitosterol (Table 3). The IH^∗^ extract also revealed nine peaks, including hexadecanoic acid, methyl ester, and β-sitosterol (Table 4). The OJ^∗^ extract demonstrated 14 peaks, with compounds like auraptenol, osthole, and β-sitosterol, among others (Table 5). The major chemical constituents of the AP^∗^ extract were identified as methyl tetradecanoate (41.19%, [49]) and β-sitosterol (21.85%, [50]), known for antitumor, antioxidant, and antimicrobial activities [43, 45, 47]. IH^∗^ extract contained β-sitosterol (43.33%), octacosyl trifluoroacetate (12.10%), and hexadecanoic acid, methyl ester (10.82%) and has cytotoxic [50], antioxidant [52], and antimicrobial activities [44, 45]. OJ^∗^'s major compound was auraptenol (58.46%), which exhibits antidepressant [68] and antiproliferative effects [69], followed by osthole (15.36%), which has anticancer, anti-inflammatory, and antidiabetic activities [63–65], and pabulenol (6.79%), known for platelet anti-aggregatory and antioxidant effects [71, 72]. Shared compounds among all three herbs—β-sitosterol and hexadecenoic acid, methyl ester—have demonstrated cytotoxic, antioxidant, and antimicrobial activities. These findings support the traditional use of these Thai herbs for treating anti-inflammatory disorders.

## 4. Conclusion

AP, IH, and OJ are commonly used in Thai Traditional Medicine to enrich women's blood and treat various female-specific health issues. Our scientific findings confirm their antioxidant properties and cytotoxic effects against breast cancer cell lines. Notably, ethanol extracts of AP and OJ exhibited significant antiestrogenic activity. Although OJ exhibited lower antioxidant activity, it showed selective cytotoxicity against breast cancer cells. The chemical constituents identified in these ethanol extracts possess diverse biological activities, reinforcing their traditional use in treating women's health issues. Nevertheless, further research is needed to investigate the underlying mechanisms and assess toxicity through in vivo studies.

## Figures and Tables

**Table 1:** Antioxidant capacity assays (DPPH, FRAP, and nitric oxide).

Plant	Common name	Part used	Extraction	Antioxidant activity		
				DPPH	FRAP	Nitric oxide (NO)
				EC_50_ ± SEM (μg/mL)	(mg Trolox equivalent/g)	
*Ardisia polycephala* Wall.	Wallich's coralberry	Fruits	EtOH, maceration	10.95 ± 1.70	122.95 ± 2.01^b^	46.44 ± 0.11^b^
			Water, decoction	7.09 ± 1.47	358.52 ± 1.07^b^	72.18 ± 2.58^b^

*Iresine herbstii* Hook.	Bloodleaf	Leaf	EtOH, maceration	20.43 ± 2.81^b^	157.50 ± 1.98^b^	12.99 ± 1.22^b^
			Water, decoction	22.61 ± 1.46^b^	185.92 ± 1.46^b^	50.88 ± 3.95^b^

*Oenanthe javanica* (Blume) DC.	Water dropwort	Fruits	EtOH, maceration	> 100	17.72 ± 0.85^b^	32.30 ± 1.50^b^
			Water, decoction	34.14 ± 2.88^b^	102.90 ± 3.32^b^	45.03 ± 0.86^b^

Butylated hydroxytoluene: BHT^a^	—	—	—	13.72 ± 2.08	5.66 ± 0.26	NT

Acetaminophen: ACP^a^	—	—	—	NT	NT	99.50 ± 0.43

*Note:* Data were analyzed by one-way ANOVA and Dunnett's multiple comparison tests. A significant difference (^b^) is when *p* < 0.05 compared with the positive control (BHT) or standard drug (acetaminophen). (*n* = 3).

Abbreviation: NT, not tested.

^a^Positive control.

**Table 2:** Inhibitory activities of AP, IH, and OJ extracts against E2-enhanced MCF-7 and T47D cell proliferation.

Sample	MCF-7				T47D			
	iEqE50^a^	iEqE10^a^	iEqE1^a^	IL^b^	iEqE50^a^	iEqE10^a^	iEqE1^a^	IL^b^
AP^∗^	0.64	0.01		S^d^	0.1	3.1	3.77	S^d^
AP^∗∗^	6.92	9.97	9.98					
IH^∗^	86.41	94.80	96.69				88.55	
IH^∗∗^	0.96	0.95	0.95	S^d^				
OJ^∗^	0.34	0.12		S^d^	7.7	2.47	1.3	S^d^
OJ^∗∗^	1.94	0.29		M^c^				
Tamoxifen^e^	< 0.00001	0.016	0.031		< 0.00001	0.001	0.007	

^a^iEqE50, iEqE10, and iEqE1 represent the concentrations of the compounds (μM) that decreased cell proliferation (enhanced by 100 pM E2) to equivalent levels induced by 50, 10, and 1 pM E2 treatment, respectively. The values were calculated by linear regression analysis using four different concentrations.

^b^IL: inhibitory level of the extract.

^c^Mild inhibition (M): more than 50% inhibition with the concentration tested.

^d^Strong inhibition (S): more than 90% inhibition with the concentration tested.

^e^Positive control.

^∗^Extract for ethanol maceration.

^∗∗^Extract for water decoction.

**Table 3:** Phytochemical constituents identified in the ethanolic extracts of AP using gas chromatography–mass spectrometry (GC–MS).

SI no.	Retention time (min)	Common name	CAS	Molecular formula	MW (g/mol)	R. match	%Peak area	Use classification	Biochemistry and pharmacology	References
1	5.44	2-Ethyl-4-hydroxy-5-methyl-3 (2H)-furanone	27538-10-9	C_7_H_10_O_3_	142.1	901	3.77	Flavoring agent	—	—
2	10.89	3-Phenylpropionic acid	501-52-0	C_9_H_10_O_2_	150.1	948	1.49	Flavoring agent	Antimicrobial activity	[42]
3	12.65	2,6-Dimethoxyphenol	91-10-1	C_8_H_10_O_3_	154.1	936	12.19	Flavoring agent	Antioxidant, antimutagenic, anticancer	[43, 44]
4	28.66	9,12-Octadecadienoic acid	2197-37-7	C_18_H_32_O_2_	280.4	913	1.30	Flavoring agent	Antitumor	[45]
5	28.77	9-Octadecenoic acid, ethyl ester	6114-18-7	C_20_H_38_O_2_	310.5	901	1.16	Fatty esters	Anti-inflammatory	[46]
6	29.25	Hexadecanoic acid, methyl ester	112-39-0	C_17_H_34_O_2_	270.5	954	15.36	Flavoring agent	Antimicrobial activity	[47]
7	29.86	Caryophyllene oxide	1139-30-6	C_15_ H_24_O	220.3	910	1.35	Flavoring agent	Anticancer, antioxidant, antimicrobial	[48]
8	35.95	Methyl tetradecanoate	124-10-7	C_15_H_30_O_2_	242.4	926	41.49	Flavoring agent	Antioxidant	[49]
9	38.21	β-Sitosterol	83-46-5	C_29_ H_50_O	414.7	901	21.85	Fragrance ingredient	Cytotoxic	[50]

*Note:* — means not detected. %peak area to that the known compounds described by the National Institute of Standards and Technology (NIST) library. SI No., serial number.

Abbreviation: CAS, chemical abstract service.

**Table 4:** Phytochemical constituents identified in the ethanolic extracts of IH using gas chromatography–mass spectrometry (GC–MS).

SI no.	Retention time (min)	Common name	CAS	Molecular formula	MW (g/mol)	R. match	%Peak area	Use classification	Biochemistry & pharmacology	References
1	25.47	Hexadecanoic acid, methyl ester	112-39-0	C_17_H_34_O_2_	270.5	955	10.82	Flavoring agent	Antimicrobial	[47]
2	35.62	Hexacosane	630-01-3	C_26_H_54_	366.7	963	7.25	Found in many natural products	Antimicrobial	[51]
3	39.46	Octacosyl trifluoroacetate	79392-43-1	C_30_H_57_F_3_O_2_	506.8	937	12.10	Found in many natural products	Antioxidant, antimicrobial	[52]
4	39.82	Tetradecanoic acid	544-63-8	C_14_H_28_O_2_	228.37	907	3.02	Flavoring agent	DMPC and PMPC inhibit SARS-CoV-2 infection in cells	[53]
5	40.59	Supraene	111-02-4	C_30_H_50_	410.7	905	3.43	Found in many natural products	—	—
6	41.11	1,16-Hexadecanediol	7735-42-4	C_16_H_34_O_2_	258.4	924	3.07	Found in many natural products	Antifungal, anti-inflammatory, antinociceptive	[54–56]
7	41.27	Octacosanol	557-61-9	C_28_ H_58_O	410.8	915	9.83	Found in many natural products	Cholesterol-lowering effects	[57]
8	42.74	(Z)-14-Tricosenyl formate	—	C_24_H_46_O_2_	366.6	915	7.16	Found in many natural products	—	—
9	45.42	β-Sitosterol	83-46-5	C_29_ H_50_O	414.7	902	43.33	Fragrance ingredient	Cytotoxic	[50]

*Note:* — means not detected. %peak area to that the known compounds described by the National Institute of Standards and Technology (NIST) library. SI No., serial number.

Abbreviation: CAS, chemical abstract service.

**Table 5:** Phytochemical constituents identified in the ethanolic extracts of OJ using gas chromatography–mass spectrometry (GC–MS).

SI no.	Retention time (min)	Common name	CAS	Molecular formula	MW (g/mol)	R. match	%Peak area	Use classification	Biochemistry and pharmacology	References
1	25.03	Limonen-6-ol, pivalate	—	C_15_H_24_O_2_	236.3	932	0.60	Found in many natural products	Antimicrobial, ameliorative effects	[58–60]
2	25.47	Hexadecanoic acid, methyl ester	112-39-0	C_17_H_34_O_2_	270.5	955	1.68	Found in many natural products	Antimicrobial	[47]
3	27.79	Methoxsalen	298-81-7	C_12_H_8_O_4_	216.1	958	4.56	For the treatment of psoriasis and vitiligo	Relieve hyperproliferative skin diseases	[61]
4	28.52	1-Eicosanol	629-96-9	C_20_H_42_O	298.5	931	0.58	Used as an emulsion stabilizer and viscosity-increasing agent		[62]
5	29.26	Methyl tetradecanoate	124-10-7	C_15_H_30_O_2_	242.4	958	1.36	Flavoring agent	Antioxidant	[49]
6	29.52	Osthole	484-12-8	C_15_H_16_O_3_	244.2	964	15.36	Found in many natural products	Anticancer, anti-inflammatory, antidiabetic	[63–65]
7	31.17	Isomeranzin	—	C_15_H_16_O_4_	260.2	927	0.73	Found in many natural products	Anti-inflammatory, antiviral (herpes simplex virus)	[66, 67]
8	31.29	2,3-Dihydro-9-hydroxy-7H-furo [3,2-g][1] benzopyran-7-one	68123-30-8	C_11_H_8_O_4_	204.1	930	6.74	Found in many natural products	—	—
9	32.15	Auraptenol	1221-43-8	C_15_H_16_O_4_	260.2	964	58.46	Found in many natural products (coumarin compound)	Antidepressant, antiproliferative	[68, 69]
10	33.80	Ethyl arachidate	18281-05-5	C_22_H_44_O_2_	340.6	913	0.65	Found in many natural products	Anti-inflammatory and analgesic	[70]
11	36.54	Pabulenol	33889-70-2	C_16_H_14_O_5_	286.2	769	6.79	Found in many natural products (coumarin compound)	Platelet antiaggregatory effects, antioxidant	[71, 72]
12	44.41	Campesterol	474-62-4	C_28_H_48_O	400.7	948	0.41	Found in many natural products	Antiangiogenic	[73]
13	44.67	Stigmasterol	83-48-7	C_29_H_48_O	412.7	957	0.66	Found in the fats and oils of soybean	Antiosteoarthritic, thyroid inhibitory, antiperoxidative hypoglycemic effects	[74, 75]
14	45.41	β-Sitosterol	83-46-5	C_29_H_50_O	414.7	978	1.42	Fragrance ingredient	Cytotoxic	[50]

*Note:* — means not detected. %peak area to that the known compounds described by the National Institute of Standards and Technology (NIST) library. SI No., serial number.

Abbreviation: CAS, chemical abstract service.

## Data Availability

The experimental datasets conducted during the current study are available from the corresponding author upon reasonable request.

## Nomenclature

AP: *Ardisia polycephala*
IH: *Iresine herbstii*
OJ: *Oenanthe javanica*
TTMs: Thai Traditional Medicine
GC–MS: Gas chromatography–mass spectrometry
DPPH: 2, 2-Diphenyl-1-picrylhydrazyl
^∗^: Ethanolic maceration
^∗∗^: Water decoction
ACP: Acetaminophen
BHT: Butylated hydroxytoluene
FRAP: Ferric reducing antioxidant power
NO: Nitric oxide
DMSO: Dimethylsulfoxide
PBS: Phosphate-buffered saline
MTT: Thiazolyl blue tetrazolium bromide
IC50: Inhibition concentration at 50%
MEM: Minimum essential medium
RPMI: Rosewell Park Memorial Institute
FBS: Fetal bovine serum
NEAA: Nonessential amino acid
P/S: 50 IU/mL penicillin, 50 μg/mL streptomycin
TCA: Trichloroacetic acid, [Cl3CCOOH]
SRB: Sulforhodamine B sodium salt, [C27H29N2NaO7S2]
E2: Estradiol
DCC: Dextran-coated charcoal
iEqE: Inhibits the growth of cells enhanced by 100 pM of E2 to the equivalent of those induced
NIST: National Institute of Standards and Technology
NT: Not tested
